# Kinematics in the brain: unmasking motor control strategies?

**DOI:** 10.1007/s00221-017-4982-8

**Published:** 2017-06-02

**Authors:** Liesjet E. H. Van Dokkum, D. Mottet, I. Laffont, A. Bonafé, N. Menjot de Champfleur, J. Froger, E. Le Bars

**Affiliations:** 1 Laboratoire Charles Coulomb, University of Montpellier, 34095 Montpellier, France; 2 Division of Interventional Neuroradiology, I2FH, Gui de Chauliac, Montpellier University Hospital, 34295 Montpellier, France; 3 Euromov, University of Montpellier, 34090 Montpellier, France; 4 Physical Medicine and Rehabilitation, Lapeyronie, Montpellier University Hospital, 34295 Montpellier, France; 5 Physical Medicine and Rehabilitation, Le Grau du Roi, Nimes University Hospital, 30240 Nimes, France

**Keywords:** Motor control, fMRI, Kinematics, Neural networks, Error corrections, Upper limb

## Abstract

In rhythmical movement performance, our brain has to sustain movement while correcting for biological noise-induced variability. Here, we explored the functional anatomy of brain networks during voluntary rhythmical elbow flexion/extension using kinematic movement regressors in fMRI analysis to verify the interest of method to address motor control in a neurological population. We found the expected systematic activation of the primary sensorimotor network that is suggested to generate the rhythmical movement. By adding the kinematic regressors to the model, we demonstrated the potential involvement of cerebellar–frontal circuits as a function of the irregularity of the variability of the movement and the primary sensory cortex in relation to the trajectory length during task execution. We suggested that different functional brain networks were related to two different aspects of rhythmical performance: rhythmicity and error control. Concerning the latter, the partitioning between more automatic control involving cerebellar–frontal circuits versus less automatic control involving the sensory cortex seemed thereby crucial for optimal performance. Our results highlight the potential of using co-registered fine-grained kinematics and fMRI measures to interpret functional MRI activations and to potentially unmask the organisation of neural correlates during motor control.

## Introduction

During rhythmical movement, sensory and motor systems need to interact closely to sustain the rhythm and to meet task requirements. Understanding how our system controls such a basic, all day movement is a prerequisite to improve motor (re)learning models to ameliorate rehabilitation in case of neurological movement disorders, like stroke. Mathematically, the simplest way to model rhythmicity is by means of a continuous oscillator (e.g. Haken et al. 1985). Biological noise interfering with planning and execution makes human movements unavoidably variable, which asks for correction processes (Franklin and Wolpert 2011). One of the principles governing human motor control states that optimised control is characterised by a maximum efficiency, e.g. minimal costs (Guigon et al. 2007). Minimal cost is dependent on the varying interaction between different system characteristics, including anatomical constraints, force generating capacities, and biological noise inducing the intra and interpersonal variability that is inherent to our system’s output (van Galen and Hueygevoort 2000).

Current knowledge about the neural correlates of rhythmical upper limb movement is based on standard finger and wrist movement paradigms that compare different movement conditions within people (high frequency versus low frequency, Kelso et al. 1998; rhythmic versus discrete movements, Schaal et al. 2004). Using this paradigm, simple unilateral rhythmical movements have been shown to elicit contralateral activations of the primary sensorimotor cortex (S1 + M1) and of the supplementary motor area (SMA), complemented by an ipsilateral activation of the anterior cerebellum (Allison et al. 2000; Ball et al. 1999; Schaal et al. 2004). Bilateral movements are associated with a symmetric facilitation of neural activity in the sensorimotor network, with additional frontal activations to ensure coordination between limbs. It is mediated by increased intrahemispheric connectivity and enhanced transcallosal coupling of SMA and M1 (Grefkes et al. 2008; Jäncke et al. 2000).

The activation pattern is comparable between dominant and non-dominant sided movements in extension and intensity when people move at their preferred frequency (Lutz et al. 2005; Jäncke et al. 2000; Koeneke et al. 2004). However, when movement frequency is imposed, activations during non-dominant sided movements increase in intensity compared to those of the dominant side (Lutz et al. 2005). Second, activation increases and expands for both uni and bilateral movements when movement frequency is increased above the preferred frequency (e.g. Kelso et al. 1998; Rao et al. 1996). Together, this demonstrates that moving at a non-preferred frequency is marked by an increase in costs. Therefore, imposing a fixed frequency may lead to different task-induced cost levels between participants and thus lead to biased results when comparing rhythmical motion and its neural correlates between people.

Over the time course of the movement, fine-grained kinematic variables capture the outcome of the interaction between the planned movement and the noise-dependent variability (Newell and Corcos 1993). Here, we explored whether kinematics may additionally provide information on the underlying control system, when the kinematic outcome is linked directly to brain activity. We simultaneously recorded brain activation (fMRI) and movement kinematics during a sensorimotor task that consisted of a self-paced continuous flexion/extension of the elbow. We focused on uni as well as bilateral movements, as many daily living tasks involve bilateral coordination. The task is evaluated as a simple well-known movement that does not require complex motor learning.

Based on the described theoretical model of motor control, we hypothesised that rhythmic voluntary flexion of the elbow is modulated by neural networks involved in (1) the sustained execution of the basic oscillatory rhythmical component and (2) correction processes in reaction to the variability resulting from biological noise. Sustaining the movement in rhythmical motion has been shown to involve the primary sensorimotor network, whereas discrete movements solicit additional higher cortical planning areas (Schaal et al. 2004). First, we expected to confirm the role of the sensorimotor network by performing a standard general linear-model analysis. Second, because task costs were as much equalised over participants as possible, we expected that correlating natural variation in movement execution with variation in BOLD-activation might unmask different brain regions involved in the secondary correction processes that could be (partly) separated from the primary sensorimotor network.

## Materials and methods

### Participants

Thirteen healthy volunteers (age 44.8 ± 14.5, 7 male) participated. All were right-handed (Edinburg Handedness Inventory, Oldfield 1971). The study protocol was registered as clinical trial (NCT01554449) and approved by the local ethics committee. The procedures complied with the ethical standards outlined by the Declaration of Helsinki. All participants gave written consent before inclusion. The study is part of a larger protocol focussing on rehabilitation of the upper limb early post-stroke (MARGAUT: 2010-A00596-33). These volunteers are included in the control group of the MARGAUT study. Exclusion criteria were as follows: (1) age >18 years, (2) history of neurological, psychiatric or orthopaedic disease, (3) medical treatment impacting the nervous system, (4) substance use, and (5) contra-indications to MRI. One male participant had to be excluded due to fMRI artefacts.

### Protocol

During the acquisition of functional MRI (fMRI) imaging, participants performed a continuous flexion/extension movement of the elbow with the right upper limb, the left upper limb, and the two upper limbs synchronously (bilateral). Each movement condition (e.g. right, left, and bilateral) was performed during a separate fMRI acquisition. All three experimental fMRI acquisitions followed the same time course. The order of acquisitions was fixed: participants started with the right-sided condition, followed by the left sided and finished with the bilateral condition. Participants had to close their eyes to limit interference with eye movements and to increase concentration on task realisation. The atypical selection of movement, the fixed order of execution, and the closing of the eyes were based on a pilot evaluation with an early post-stroke group for the MARGAUT protocol. First, the rather proximal elbow flexion/extension was selected, because recovery of movement post-stroke, generally, evolves from proximal to distal (Langhorne et al. 2009). Second, the order was fixed because an important effect of fatigue was observed when post-stroke participants had to perform the movement twice consecutively with their paretic upper limb. And third, closing the eyes seemed to help post-stroke subjects to better concentrate on movement realisation. Together, this protocol may allow us to study the evolution of upper limb motor recovery in an early post-stroke population. We first, however, verified the interest of the method within a healthy population as described in this work. Keeping a fixed-order acquisition for the control group allowed us to evaluate a possible order effect that could influence results. To limit the task-dependent increased risk of head movements, the participants’ head was fixed using supportive foam-blocks. Exclusion criteria for head movements were set at 1.5 mm absolute pitch, roll or jaw displacement.

A 1.5 T whole-body MRI system (MAGNETON AVANTO, Siemens, Erlangen, Germany), equipped with a standard 12-channel receive-only head coil, was used. Sixty volumes of BOLD echo-planar image (EPI) were obtained for each fMRI acquisition. Each acquisition followed a block-design of a three-times repeated sequence of ten ‘resting’ volumes alternated with ten ‘active’ (flexion/extension movement of the elbow) volumes (TR = 3.56 s, TE = 50 ms, FOV = 230 mm, 36 axial slices extending from the vertex to lower parts of the cerebellum, slice gap = 0.75 mm, voxel-size 3.59 × 3.59 × 3 mm^3^, flip angle = 90°). The start of each block (duration 35.6 s) was announced by a rapid auditory stimulus (500 ms). A 3DT1 MPRAGE (TR/TE/TI 2100/4.1/1100ms, 15° flip angle, aligned with the corpus callosum, voxel-size 0.98 × 0.98 × 1 mm, 176 transversal slices) was also obtained for each participant.

**Fig. 1:**
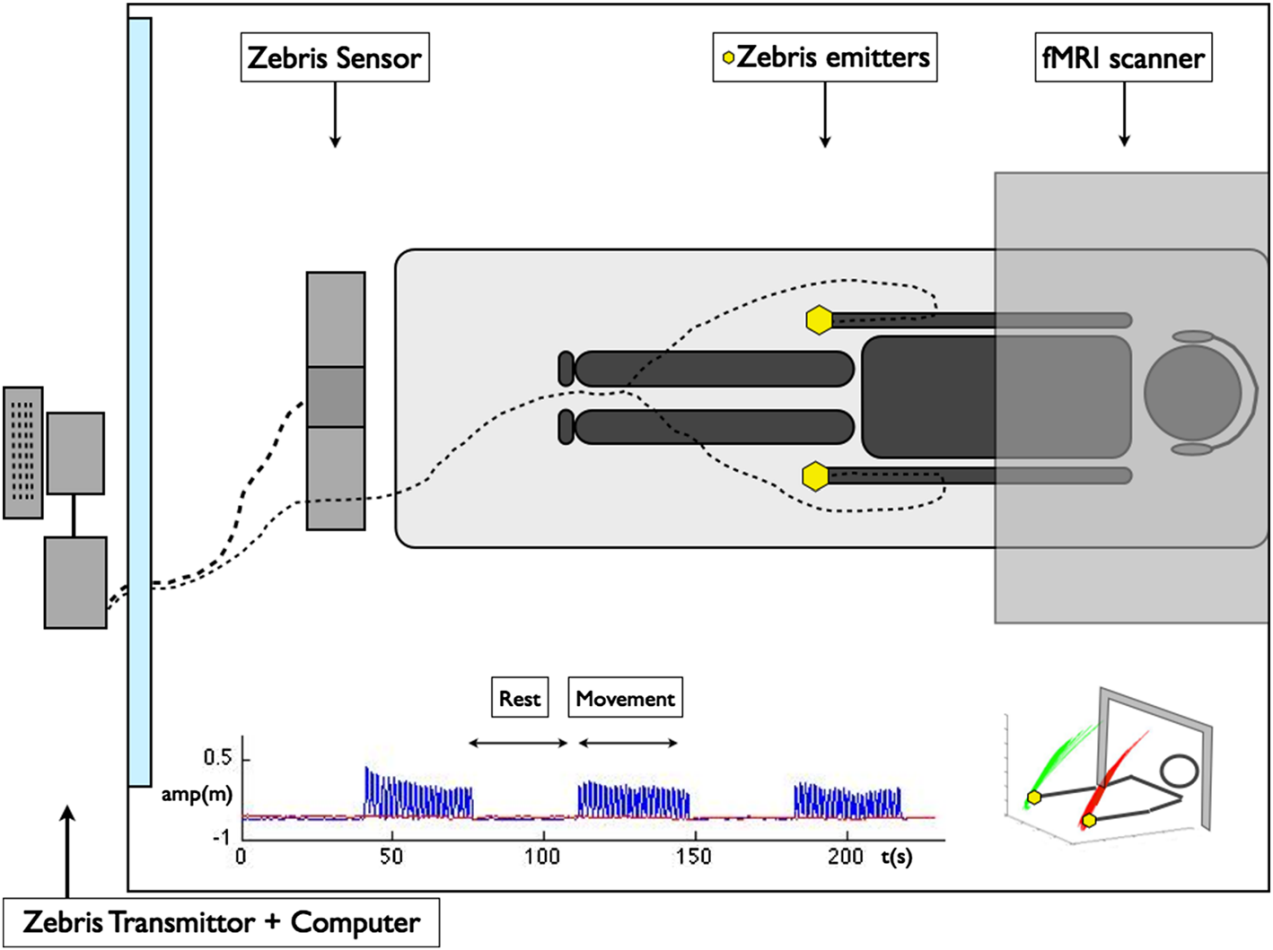
Schematic representation of experimental setup, with a plot of the* right elbow* movement amplitude during the fMRI acquisition over time (*left lower corner*) and a 3D representation of movement in space (*right lower corner*)

During rest-periods, the arms lay alongside the body with the forearm in neutral position between supination and pronation. The hands were clenched lightly in a fist. During movement-periods, the forearm and hand remained in this basic orientation while flexing and extending the elbow in a rhythmic manner, starting with the arm in the extended resting position. The maximum flexion amplitude was constrained by the MRI tube. Participants were free to select a comfortable amount of flexion within this boundary. Participants practised the procedures in fMRI setting before onset. They were instructed to relax, to move nothing but the elbow joint, and especially to limit head movements. The performed movement was registered using a 1.5 T MRI compatible 3D motion capture system (Zebris^®^, CMS-20, Medical GmbH, Isny, Germany). A marker was placed on the head of the third proximal phalange of each hand. Data were low-pass filtered at 5 Hz with a zero-lag Butterworth filter. The Zebris^®^ receiver was placed in front of the fMRI tube at the end of the scanner table (180 cm) (Fig. 1). The quality of the fMRI recordings, and the assumed compatibility between the Zebris^®^ and the MAGNETON AVANTO 1.5 T MRI, was evaluated before study onset. No interference and artefacts on fMRI images were observed when the Zebris’s^®^ sampling frequency was set at 20 Hz.

### Kinematic preprocessing

Simple oscillatory movements typically present a smooth and bell-shaped velocity profile that is slightly variable among cycles (Mottet and Bootsma 1999), signing the presence of multiple time scales in the control process (Newell and Corcos 1993; Ramdani et al. 2009). The most important aspects of movement organisation pertain to its scaling, shaping (Mottet and Bootsma 1999), and the structure of its variability (Sethi et al. 2013). To capture these aspects and gain insight into the organisation of the movement, two groups of kinematic variables were selected: (1) shaping kinematics and (2) structural kinematics. To calculate these, we extracted the principal oscillation using a principal component analysis with *x*, *y*, *z* time series of hand position as input. Then we parsed the principal oscillation in sub-cycles of one flexion and extension movement based on the maxima between two velocity peaks.

### Kinematic data analysis

First, the shape of the movement was defined by calculating the frequency (FREQ: how fast) and the amplitude (AMP: how large) of the principal oscillation. We calculated the absolute distance between the lowest and highest point of each sub-cycle and defined the mean of all sub-cycles to get AMP. The total number of cycles within time was then used to calculate the FREQ of the principal oscillation.

Second, three dimensionless variables were calculated to define the structure of the movement: the smoothness of the movement, the directness of the trajectory in space, and the irregularity of the movement’s structure orthogonal to the principal oscillator. These variables provided more detailed information on the control strategy used. The smoothness of the movement was quantified by counting the number of velocity peaks (NVP) on the *x*, *y*, *z* time series. As the movement was self-paced and spatially not restricted, the NVP was corrected for the FREQ and the AMP. The higher the NVP value, the less smooth was the unfolding of the trajectory in space–time, which indicates a higher amount of submovements towards the goal (Todorov 2004). The directness of the trajectory in space was defined by the normalised trajectory length (nTL). By normalising the movement time series by its standard deviation in space, differences in path length represent the ‘deviations’ around the optimal trajectory rather than the actual trajectory length in the 3D space (Donker et al. 2008). The higher the nTL value, the longer was the hand-path trajectory between flexion and extension. It provides information on the amplitude of the disparity between the optimal and the actual trajectory.

Finally, to gain insight into the structure of the variability of the movement, the deviation from the average ideal oscillation as the Cartesian distance to the origin (Strang et al. 2012) was computed on the plane orthogonal to the principal oscillator. The irregularity of the variability was calculated using a sample entropy measure (SampEn) (Ramdani et al. 2009), following the algorithm from PhysioNet, with *M* = 2 and *r* = 0.2 (Richman and Moorman 2000). The higher the SampEn value, the higher was the irregularity of the variability around the main oscillation, which has been interpreted as a sign of automatic control (Roerdink et al. 2006). Notably, in the domain of motor control the SampEn has mainly been used in relation to postural control. Consequently, the generalisation of a rationale to the control of another type of behaviour has to be made carefully. Postural oscillations seem to be the natural consequence of intermittent open loop actions to counteract the unstable nature of the bipedal posture (Loram and Lakie 2002; Loram et al. 2011). To prevent falls in an economical fashion, posture is intermittently stabilised with serial ballistic impulses towards the goal of the task: standing still. Despite the obvious differences between the tasks of standing still and of rhythmically flexing the elbow (Craik 1947; Loram et al. 2005; Vince 1948), the mode of control shows some similarities. To prevent end-point (the hand) deviations from the preferred trajectory around the fixed point (the elbow) in an economical fashion, the elbow ‘oscillation’ trajectory will be intermittently stabilised by means of serial ballistic impulses towards the goal of the task: flexing/extending the elbow, while preventing internal/external rotational movements. This rationale guided the applied data analysis: the way we calculated SampEn was not based on the primary voluntary oscillation, but the captured irregularity of the variation along the main oscillation, i.e. the corrections of the continuous falls out of the goal trajectory.

To sum up, the NVP captures the amount of submovements during the oscillation; the nTL captures the amplitude of the directional variability of the hand-path trajectory in space; And the SampEn captures the irregularity of this variability in the plane orthogonal to the principal oscillator independent of its amplitude; i.e. the automaticity of control. The kinematic variables are mathematically independent and are normally distributed. Differences between left, right, and bimanual movements for each kinematic variable were evaluated with repeated measures ANOVA. Correlation between kinematics was evaluated by means of a simple Pearson’s correlation matrix.

### fMRI data analysis

Spatial preprocessing was performed using SPM8 (http://www.fil.ion.ucl.ac.uk/spm/) and MATLAB (R2012a, The MathWorks). Images were re-oriented to the anterior commissure, slice-time corrected, realigned to the first volume, co-registered, normalised to T1 template (provided by Montreal Neurological Institute MNI), and smoothed (8 mm FWHM Gaussian filter).

For each experimental condition, a general linear model analysis was performed based on the time-course model of the BOLD-response function in relation to the stimuli onset, to define brain activations related to the motor task. Realignment parameters were included in the model. Statistical *t*-maps comparing the active blocks with resting blocks were generated on a voxel-by-voxel basis. Contrast between ‘active’ and ‘rest’ was then calculated per participant per condition. The main effect of task was analysed by means of a full factorial analysis [family wise error corrected (FWE), *p* < 0.05]. A within-subject ANOVA was performed to gain insight into the specific condition effects (*T* threshold: *p* < 0.001, FWE corrected at cluster level).

Brain activation was evaluated against specific movement characteristics by adding the individual kinematic variables as linear regressors into the design matrix for each specific participant, describing the evolution of brain activity (intensity of activation) against variation in kinematic variables (the actual movement executed) over participants. An explorative low cluster-forming *T* threshold for peak voxel activation was used (uncorrected *p* < 0.05), with a cluster size >10 voxels.

The AAL toolbox was used to identify the clusters via local maximum analysis based on peak activation values (Tzourio-Mazoyer et al. 2002). Additionally, the percentage of the identified region covered by the cluster was evaluated.

## Results

### Participants

There was no correlation between the kinematic variables and the age of the subjects (AMP/age *p* = 0.84, *r* = −0.06; FREQ/age *p* = 0.61, *r* = 0.16; NVP/age *p* = 0.062, *r* = −0.55; nTL/age *p* = 0.16, *r* = −0.43; SampEn/age *p* = 0.12, *r* = 0.46), nor did we find an impact of age on brain activations.

### Movement kinematics

Flexion/extension of the elbow is a well-known movement in the context of rehabilitation. The elbow movement was associated with a fluid, rhythmic motion that was well integrated in the corporeal capacity. Its velocity profile had a smooth sinusoidal appearance with slight variations between cycles resulting from natural noise. No outliers were identified and no significant differences between right, left or bilateral movements were observed for any of the kinematic variables. Overall, participants scaled their movement in time and space with a mean FREQ of 0.63 Hz (±0.17 Hz) and AMP of 33.2 cm (±6.18 cm). Their movement was structured in time and space with an average NVP of 40.1 (±3.7), nTL of 1.11 (±0.34), and SampEn of 1.17 (±0.29). Correlation between kinematic variables can be found in Table 1. A positive correlation is observed between FREQ and SampEn (*r* = 0.78, *p* = 0.0096) and a negative correlation between nTL and SampEn (*r* = −0.45, *p* = 0.0034).

**Table 1:** *r* values of correlation matrix between kinematics

	AMP	FREQ	NVP	nTL	SampEn
AMP	1				
FREQ	−0.39	1			
NVP	0.25	−0.38	1		
nTL	−0.40	0.05	0.00	1	
SampEn	−0.24	0.78*	−0.37	−0.45*	1

*AMP* amplitude, *FREQ* frequency, *NVP* number of velocity peaks, *nTL* normalised trajectory length, *SampEn* sample entropy

* Significance at *p* < 0.01; significant level between FREQ and SampEn *p* = 0.0096 and between nTL and SampEn *p* = 0.0034

### Functional basis network

No movement artefacts were observed during the task for the 12 volunteers. The movements of the head were between a maximum average translation of 0.74 mm and a maximum average rotation of 0.016° and a minimum average translation of −1.08 mm and a minimum mean rotation of −0.0096°.

**Fig. 2:**
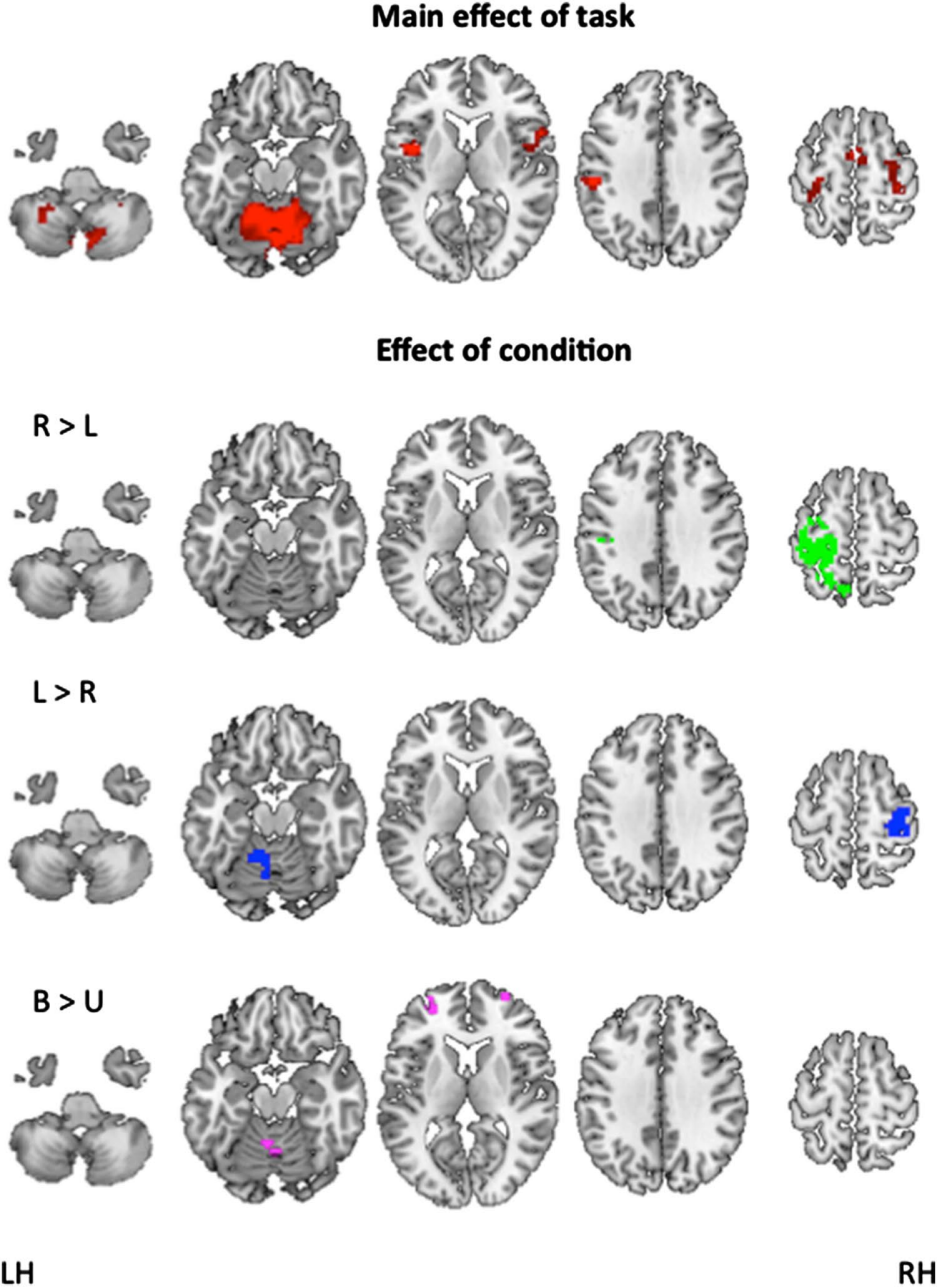
Functional basis network: the main effect of task (flexion/extension of the elbow), FWE corrected, *p* < 0.05 at voxel level and the condition-specific activations *p* < 0.001, FWE corrected at cluster level, 22 degrees of freedom. *R* right sided, *L* left sided, *B* bilateral, *U* unilateral movement, *RH* right hemisphere, *LH* left hemisphere

As expected, the continuous flexion/extension of the elbow elicited, primarily, the activation of the sensorimotor network including the primary motor cortex, the primary sensory cortex, and the supplementary motor area (SMA). It also elicited activations of the supramarginal gyrus and the rolandic operculum. The within-subject ANOVA showed that the left-sided primary sensorimotor cortex was specifically linked to right-sided movement, as was the right-sided primary sensorimotor cortex to left-sided movement. Bimanual movements were characterised by additional activations of the vermis and of the middle frontal regions, which are known to play an important role in motor planning when performing bimanual tasks (Jäncke et al. 2000). A detailed overview of task and condition activations can be found in Table 2 and Fig. 2.

**Table 2:** *x*, *y*, *z* MNI coordinates of the peak voxel in relation to cluster size (*K*_E_) and *F* score [*F*] for the main effect of task (FWE corrected at voxel level, *p* < 0.05, 33 degrees of freedom), cluster size, and *T* score [*T*] for condition-specific activations (*p* < 0.001, FWE corrected at the cluster level, 22 degrees of freedom), and defined with AAL toolbox SPM8

Region	RL	Main task activation					Right > left					Left > right					Bimanual > unimanual				
		*x*	*y*	*z*	*k*_E_	[*F*]	*x*	*y*	*z*	*k*_E_	[*T*]	*x*	*y*	*z*	*k*_E_	[*T*]	*x*	*y*	*z*	*k*_E_	[*T*]
Primary motor cortex	R	30	−40	66	57	56						34	−17	63	110	4.75					
	L	−29	−27	66	−27	54	−29	−23	63	454	7.62										
Primary sensory cortex	R	34	−30	63	57	44						34	−30	63	110	6.66					
	L	−32	−37	51	−37	55	−35	−30	63	454	7.62										
Supplementary motor area	R	7	3	51	144	76															
	L	−9	6	40	144	40															
Middle frontal area	R																27	59	14	30	4.23
	L																−29	55	14	42	4.79
Rolandic operculum	R	56	13	6	41	68															
	L	−42	−0	14	44	91															
Supramarginalis	R	60	−30	25	30	72															
	L	−55	−23	40	89	94															
Cerebellum	R	27	−46	−27	1171	226															
	L	−18	−50	−24	1171	226						−12	−46	−20	61	5.25					
Vermis		1	−63	−20	1171	199											−1	−66	−20	54	5.28

Note that we found a more extended activation of the primary sensorimotor network during dominant right-sided movements. This was surprising, as participants moved at a self-selected speed, which should induce comparable levels of effort between dominant and non-dominant sided movements, and thus comparable levels of extension and intensity of activation. This might indicate the presence of an order effect, resulting from the fixed order of task execution (right, left, and bimanual).

### Kinematic related functional network

First, we executed a separate linear regression with a low cluster-forming *T* threshold for each movement condition (left/right/bimanual) to explore the link between the brain activation and the kinematics of a movement. We observed two phenomena: (1) there were condition-specific differences, dependent on whether the movement was performed with the dominant, non-dominant or both sides, and (2) there were general similarities in activation patterns independent on movement condition (Table 3). Based on these observations, we hypothesised that these general similarities might indicate the existence of common task-dependent control strategies that are independent of condition-specific activations.

**Table 3:** Kinematic related activations

	Right			Left			BIM		
	Zone	[*x*, *y*, *z*]	*K*_E_, [*T*]	Zone	[*x*, *y*, *z*]	*K*_E_, [*T*]	Zone	[*x*, *y*, *z*]	*K*_E_, [*T*]
AMP									
G	Front inf R	[30, 29, −9]	23, 2.79	Temp sup L	[−45, −7, −5]	58, 5.04	Front inf L	[−55, 9, 33]	65, 3.70
	Front inf L	[−35, 36, 14]	13, 5.08	Temp mid R	[56, −10, −9]	25, 2.90	Temp mid L	[−65, −56, 6]	53, 3.39
	Temp mid R	[37, 9, −35]	11, 2.38				Vermis 45	[1, −53, −6]	43, 3.77
	Vermis 45	[17, −46, 10]	60, 4.78						
S	Precun L	[−6, −63, 29]	39, 3.73	Amygdala R	[20, 0, −16]	41, 5.03	Ant cerebellum R	[7, −37, −9]	37, 4.39
	Occip mid R	[40, −86, 21]	15, 3.76	Insula R	[43, 0, 3]	63, 4.31			
FREQ									
G	Post cerebellum L	[−26, −69, −24]	20, 3.77	Post cerebellum L	[−16, −82, −35]	28, 4.85	Post cerebellum L	[−48, −69, −20]	20, 4.83
S	Hippocamp L	[−26, −20, −13]	12, 4.24	Front sup R	[11, 26, 40]	175, 5.85	Post central L	[−32, −30, 51]	127, 4.79
	Temp sup L	[−65, −50, 18]	42, 4.14	Front sup L	[−22, 36, 51]	39, 4.99	Angular R	[43, −60, 40]	12, 4.03
	Temp sup R	[60, −50, −1]	11, 3.49	Front mid L	[−32, 42, 18]	39, 4.75	Cing ant RL	[7, 26, −9]	12, 3.98
NVP									
G	Front mid R	[40, 42, 3]	37, 3.41	Supramarg R	[60, −17, 18]	50, 5.48	Front mid R	[30, 6, 51]	81, 11.29
	Supramarg R	[66, −23, 33]	89, 4.16	Temp mid R	[43, −60, 6]	113, 5.54	Front mid L	[−26, 6, 48]	83, 6.13
	Temp mid R	[40, −63, 14]	41, 2.77	Cing mid R	[24, −10, 59]	186, 4.94	Supramarg R	[56, −23, 18]	59, 5.61
	Cing mid L	[−6, 3, 44]	35, 3.52				Temp mid L	[−62, −6, −5]	15, 5.27
S				Fusiform L	[−35, −46, −20]	53, 6.00			
				SMA R	[4, −0, 36]	186, 4.80			
				Lingual R	[14, −60, −20]	103, 3.93			
nTL									
G	Temp mid L	[−48, −14, −24]	50, 4.42	Temp mid R	[43, −73, 18]	49, 6.81	Temp mid L	[−45, −14, −28]	49, 3.75
	Parahipp L	[−22, −14, −28]	27, 3.07	Parahipp R	[20, −46, −1]	16, 4.21	Parahipp R	[24, 0, −24]	39, 4.25
							Parahipp L	[−16, −17, −24]	26, 3.40
S	Post central R	[53, −20, 33]	45, 3.28	Front inf op L	[−39, 9, 21]	26, 6.43	Fusiform L	[−19, −46, −16]	41, 3.67
	Cing ant R	[7, 36, −1]	33, 3.22	Cing ant R	[7, 26, 25]	19, 3.55			
				Lingual L	[−22, −73, −9]	17, 4.75			
SampEn									
G	Front mid R	[37, 39, 40]	36, 6.29	Front mid R	[43, 13, 36]	134, 7.49	Post cerebellum L	[−48, −69, −28]	26, 4.57
	Post cerebellum L	[−32, −46, −31]	245, 5.71	Post cerebellum L	[40, −46, −35]	32, 5.04	Post central L	[−32, −37, 55]	59, 4.35
				Post central L	[−26, −33, 63]	30, 4.09			
S	Occip mid L	[−26, −73, 29]	326, 8.98	Front sup LR	[−22, 36, 55]	759, 9.79	Precuneus R	[17, −56, 21]	121, 4.86
	Temp mid L	[−65, −50, 3]	145, 7.08	Caudate LR	[14, 13, 10]	166, 7.85			

[*x*, *y*, *z*] MNI coordinates of peak value, cluster size (*K*_E_), and *T* value [*T*] of each cluster >10 voxels related to the kinematic variables for the separate conditions (Right, Left, BIM) with an explorative low *T* threshold of *p* < 0.05, uncorrected

*L* left hemisphere, *R* right hemisphere, *G* task-general activation, *S* condition-specific activation

Aware of the increased risk of false positives when using low cluster-forming *T* thresholds (Eklund et al. 2016), we repeated the linear regression, but this time we grouped all conditions together allowing an increase in statistical power. The cluster-forming *T* threshold was increased to *p* < 0.005 uncorrected with cluster >30 voxels, visible in Table 4. We found indeed most of the general activations, but we also observed the emergence of some new regions, and the disappearance of some others with the stricter threshold applied. The correlation between the fMRI BOLD response and each kinematic variable was as follows: FREQ *r* = 0.71, *F*_(1,34)_ = 32.9, *p* < 0.0001; NPV *r* = 0.62, *F*_(1,34)_ = 21.18, *p* < 0.0001; nTL *r* = 0.66, *F*_(1,34)_ = 25.39, *p* < 0.0001, SampEn *r* = 0.70, *F*_(1,34)_ = 31.39, *p* < 0.0001. See Table 4. Unexpectedly, the correlation of brain activation with AMP did not reach cluster-forming significance at *p* < 0.005. This might be dependent on multiple factors, like body morphology (e.g. size biceps, arm length) and the fMRI tube, influencing the preferred amplitude differently over participants. In a future study, arm length should be added as a covariate to gain a more profound insight.

**Table 4:** Overview of task-general activations after grouped correlation of all conditions between kinematics and fMRI activity, with MNI coordinates [*x*, *y*, *z*] of the peak voxel, the cluster size (*K*_E_) and the *T* score [*T*] of each cluster (*p* < 0.005 uncorrected with cluster > 30 voxels, degrees of freedom = 10)

	*x*	*y*	*z*	*k*_E_	[*T*]
FREQ					
Positive					
Posterior cerebellum L	−26	66	−20	482	5.73
Precentral R	24	−23	55	62	4.13
Negative					
xx					
NVP					
Positive					
Front mid R	20	−7	59	55	4.19
Supramarg R	56	−23	21	80	3.72
Temp mid R	47	−60	10	39	3.49
Cing mid R	7	6	44	99	4.60
Negative					
xx					
nTL					
Positive					
Post central L	−48	−23	36	36	3.65
Negative					
Posterior cerebellum L	−29	−40	−31	75	3.87
SampEn					
Positive					
Posterior cerebellum L	−26	−66	−20	120	5.60
Posterior cerebellum R	37	−79	−31	66	5.01
Frontal mid R	47	29	36	40	4.86
Negative					
xx					

No negative significant correlations were observed except for the nTL with a cluster in the left posterior cerebellum

*L* left hemisphere, *R* right hemisphere, *xx* no significant correlation

**Fig. 3:**
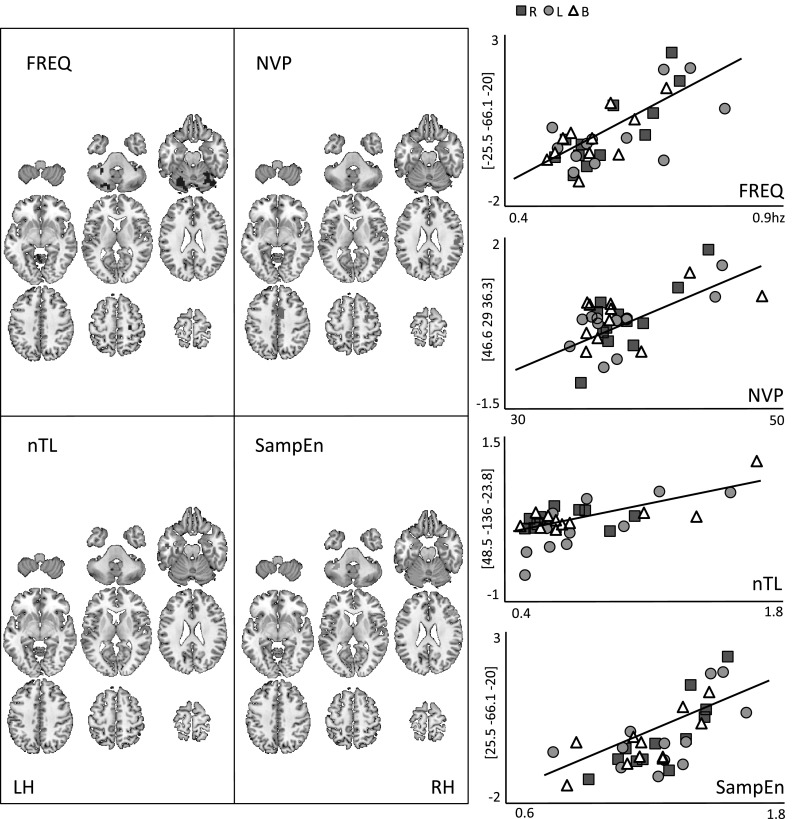
Overview of positive correlations between fMRI activity (BOLD signal intensity) and movement kinematics. *Left image* correlated clusters for the grouped movement conditions. T-contrast [0 1], *p* < 0.005, uncorrected with cluster >30 voxels, *df* = 10). *RH* right hemisphere, *LH* left hemisphere, *R* right sided, *L* left sided, *B* bilateral movement. *Right graphs* regression plots between each kinematic variable (*x-axis*) and the response intensity (*y-axis*) of the most pertinent cluster ([*x*, *y*, *z*] coordinates provided). FREQ (frequency): *r* = 0.71, *F*_(1,34)_ = 32.9, *p* < 0.0001; NPV (number of velocity peaks): *r* = 0.62, *F*_(1,34)_ = 21.18, *p* < 0.0001; nTL (trajectory length): *r* = 0.66, *F*_(1,34)_ = 25.39, *p* < 0.0001; SampEn (sample entropy): *r* = 0.70, *F*_(1,34)_ = 31.39, *p* < 0.0001

In greater detail, a stronger activation of the left posterior cerebellar lobe was observed with a higher preferred frequency, as well as increased activation of the right M1. Second, less smooth movements, as identified by a higher NVP, were linked to stronger activations of the right-sided middle frontal, supramarginal, temporal, and cingulum regions. Third, longer trajectories as identified by a higher nTL were linked to increased activation of the left S1. And fourth, a stronger irregularity of the variability around the primary oscillatory component, as identified by the SampEn, was correlated with stronger activations of both posterior cerebellar lobes and right middle frontal regions. An overview of the brain activations in relation to the kinematics and the correlation graphs can be found in Fig. 3.

We also explored the possible negative correlation between brain activity and kinematics (e.g. less activation with higher preferred frequency). It was found that only nTL was negatively correlated with the posterior cerebellum (Table 4).

## Discussion

Rhythmical movement generation involves a basic oscillatory component and correction processes to cancel out the variability induced by biological noise. Here, we explored whether looking into the variation of voluntary movement kinematics combined with the variation in brain activation might provide additional insight into actual organisation of the neural correlates of these two processes. Therefore, two analyses were performed. First, brain activation was evaluated by means of a basic general linear model, and second, kinematic characteristics of the voluntary elbow movement were added as linear regressors to the general model.

The functional basis network that was activated during elbow flexion/extension consisted of the primary sensorimotor cortex, the SMA, the supramarginal gyrus, the rolandic operculum, and the anterior cerebellum. This was comparable with motor networks described by more traditional finger-tapping paradigms (amongst others: Allison et al. 2000; Ball et al. 1999; Schaal et al. 2004; Stoodley and Schmahmann 2009). Although expected, this was an important observation, because it validated the use of the elbow flexion–extension as an alternative movement to study the link between brain activation and function. It also showed that the expected increase in task-related head movements due to the larger and more proximal nature of the movement was not sufficient to induce important artefacts. Subsequently, by adding movement kinematics as regressors to the BOLD-activation contrast matrix, two interesting observations were made: (1) only few of the regions of the functional basis network showed variations in activity with variation in movement kinematics, and (2) the activations that were observed coincided partly over conditions—right, left or bimanual.

Motor behaviour can be explained in terms of building blocks on both cerebral and spinal level that together construct the required movement (Mussa-Ivaldi and Bizzi 2000; Bizzi et al. 2002). Alongside processing conditions, specific blocks that are required to coordinate a set of muscles to perform a specific movement (Degalier and Jspeert 2010), task-general information blocks that are independent of the specific details might be processed in parallel. Based on our results, we here hypothesise that the activations that coincided over conditions might be involved in the general control of the motor task (flexion/extension of the elbow), rather than in condition-specific processes (dominant/non-dominant/bilateral task execution). Focussing on these general building blocks, we found that the irregularity orthogonal to the main oscillation, as captured by the SampEn, was related to activations of the posterior cerebellum and middle frontal regions. Tatch (1980) proposed that these regions form a network together. This cerebellar–frontal motor circuit was thought to be involved in the development and automation of internal models (Penhune and Steel 2012). The frontal regions mediate the selection of alternative motor options in a given context, whereas the cerebellum plays a crucial role in the control of the movement by means of feedforward and attention direction (Desmurget and Grafton 2000). The information from these two regions is supposed to be combined in the basal ganglia, which are thought to function as a gateway for habitual actions—like the automatic execution of routine movements (Leisman and Melillo 2012). The activation of the observed cerebellar–frontal circuit was increased with more irregular fluctuations around the principal oscillator. The irregularity of fluctuations is proposed to represent a continuity of the amount of attention invested in motor control, whereby high irregularity is considered as a signal of more automatic behavioural control, whereas more regular behaviour indicates a more conscious control of task execution (Roerdink et al. 2006).

The finding that both the network and the kinematic variable that unmasked this network have previously been linked to automaticity of control might be a coincidence. Alternatively, this finding might support the described relation between irregularity and automaticity of control as observed in postural control studies (Roerdink et al. 2006). Martinez et al. (2016) highlighted the neural correlates of stepping stability in a distributed network (subcortical/cortical region), suggesting a transition in the control strategy across the stimulated range of comfortable frequencies from more reactive (conscious, feedback) to a predictive (automatic, feedforward) control. We also found that the irregularity of the fluctuations increased with the frequency of movement. Therefore, taking our reasoning one step further, this might indicate that a higher preferred frequency reflects a more automatic control strategy, whereas a lower preferred frequency might indicate a more conscious strategy. This hypothesis, although we recognise that it is very explorative, is supported by the negative correlation between irregularity and trajectory length of the movement, as well as by the negative correlation between the posterior cerebellum with the trajectory length. The trajectory length, as captured by nTL, was positively related to activations of the primary sensory cortex—involved in sensory feedback to adjust movements based on the difference between the planned and the executed movement (Christensen et al. 2007). The higher the activations observed in the primary sensory cortex (related to more twisting and turning, i.e. a longer trajectory length), the lower was the activation in the cerebellar–frontal areas, i.e. less automatic control.

Therefore, we would like to propose with consideration that there might be various loops/circuits involved in the maintenance of self-paced comfortable rhythmic movements that are negatively correlated. The primary sensory motor network, as highlighted by the general model analysis, might be responsible for the generation of the rhythmical movement, with an additional recruitment of the middle frontal areas when coordination between sides is required in the bimanual condition. Lewis and Miall (2003) argue that there are distinct brain structures involved in tasks depending on whether these are characterised more by automatic or more by cognitive control. Automatic control primarily draws on primary motor circuits, whereas cognitive control requires additional pre-frontal and parietal areas. Subsequently, to control the neuromotor noise-induced variability that is inherent to our system, we have to our disposition a cerebellar–frontal circuit for feedforward based automatic control as well as a more feedback-based conscious control involving activation of the primary sensory cortex. The implication of each network during task execution may be dependent on the required monitoring of movement execution in time and space. The overlap of networks seems evident during more complex movements, to control movement following the logic of computational models optimally integrating feedback, with minimal effort as well as minimal error (Guigon et al. 2007).

Our long-term goal is to capitalise on a better understanding of the neural correlates of rhythmical movement kinematics to better address motor recovery post stroke. Cerebral lesions change the structure and function of the brain not only by disabling cells, or destroying normal functional brain networks, but also by unmasking, sprouting, and developing new networks (Buma et al. 2013). The whole functioning of the system may, therefore, change: relating healthy behaviour to that of an altered system may not be straightforward. When kinematics can indeed provide information about underlying functioning and organisation of control, following the change in kinematics post stroke might contribute to understanding changes in brain activation post stroke. This knowledge might be used to target specific regions for non-invasive brain stimulation protocols, modulating connectivity and eventually enhancing recovery (e.g. Ludemann-Podubecka et al. 2015). However, the first immediate challenge will be to identify how (change in) kinematics over rehabilitation is related to post-stroke neural plasticity and/or changes in activation patterns, compared to a large group of healthy controls.

Albeit these promising results, one has to bear in mind that it concerned an explorative study with a limited number of participants, that, however, confirmed the interest of the applied method, i.e. the integration of movement registration within the fMRI can contribute to a better interpretation and understanding of functional activations observed. It is known that the ageing brain operates differently compared to the young brain: over-activation of the ipsilateral motor system as well as additional recruitment has been reported frequently. Its origin and functional impact are, however, still under debate. It could point to compensation to meet task demands in the face of age-induced neurobiological changes (cognitive as well as motor) (Calautti et al. 2001), but also result from changes in cerebral perfusion during rest that are independent of the functional task demands (Riecker et al. 2006). Using a heterogeneous age group might thus have influenced the results. However, for this simple motor task this was not confirmed. This could be related to the relatively small sample size or to the experimental design. We asked middle-aged people to perform a gross motor task, rather than a young or older age group to perform a fine motor task. As pointed out by Seidler et al. (2010), it still needs to be clarified whether motor changes due to age develop gradually or show a rapid onset with older age, highlighting the importance of age mapping when comparing motor performance between different groups. Another limitation of our study is that we indeed observed an order effect, presumably resulting from the fixed-order setup. The order effect was marked by a more extended activation of the primary sensorimotor network during dominant right-sided movements, whereas normally no difference would have been expected when moving at a self-selected pace (Lutz et al. 2005). This order effect should be taken into consideration when comparing between groups and especially when addressing recovery over time. Nevertheless, the activated regions of interest were still comparable between all conditions. Therefore, we would like to argue that fixed-order effect is preferable over the effect of fatigue when moving two consecutive times with the paretic arm in a post-stroke population.

## Conclusion

Self-paced rhythmic elbow flexion/extension was a suitable task to evaluate the neural correlates of motor control. The co-registration of movement kinematics and brain activity offered the opportunity to investigate human motor control in terms of brain–behaviour relationships from a different perspective. Our results highlighted the potential of this method to interpret functional MRI activations and to take a small step towards better understanding of the organisation of neural correlates during motor control.
